# “We Feel Good”: Daily Support Provision, Health Behavior, and Well-Being in Romantic Couples

**DOI:** 10.3389/fpsyg.2020.622492

**Published:** 2021-01-18

**Authors:** Corina Berli, Philipp Schwaninger, Urte Scholz

**Affiliations:** ^1^Applied Social and Health Psychology, Department of Psychology, University of Zurich, Zurich, Switzerland; ^2^University Research Priority Program “Dynamics of Healthy Aging”, Department of Psychology, University of Zurich, Zurich, Switzerland

**Keywords:** romantic couples, support provision, provider, well-being, health behavior, accelerometer, APIM

## Abstract

Intimate partners are an important source of support when pursuing health goals. A vast amount of literature documents the role of social support in alleviating recipients’ distress and facilitating health behaviors. Less studied is the phenomenon that providing support may entail a benefit for the provider, particularly in the context of health behavior change. In the present study, we investigated whether providing social support in daily life would be associated with more health behavior, and emotional and relational well-being that same day, using a sample of romantic couples aiming to become more physically active. Ninety-nine inactive and overweight heterosexual romantic couples (=198 individuals) participated in this dyadic daily diary study. Both partners reported on the provision of social support, positive and negative affect, and relationship satisfaction in electronic end-of-day diaries across 14 consecutive days. Moderate-to-vigorous physical activity (MVPA) was objectively assessed via triaxial accelerometers (Actigraph GT3X+). Using the Actor-Partner Interdependence Model (APIM), dyadic data analyses indicated that providing support to the partner was associated with higher own MVPA, more own positive affect, less own negative affect, and more own relationship satisfaction (actor effects), over and above the effect of support provision on outcomes in the other partner (partner effects). The present findings suggest that the provision of daily social support in couples is strongly associated with enhanced well-being not only at a personal level but also at a relational level. Providing social support may also serve the function of relationship maintenance. Thus, shifting the focus away from the recipient to examine beneficial effects of social support in providers is highly relevant. Future research should address the question of when, why, and how giving support is beneficial.

## Introduction

Social relationships are widely recognized for their protective role for physical health and psychological well-being (e.g., House et al., 1988; Kawachi and Berkman, 2001; Holt-Lunstad et al., 2010). In particular, a happy romantic relationship has shown to be associated with better health outcomes (e.g., Holt-Lunstad et al., 2008). Romantic partners are an important source to turn to for help (Feeney and Collins, 2003). Social support has been proposed as one potential pathway to better health, via the facilitation of health behaviors and alleviating distress (Berkman et al., 2000). Indeed, empirical evidence suggests that support from the partner is associated with better health behaviors in recipients, e.g., daily smoking (e.g., Scholz et al., 2016; Lüscher et al., 2017), or daily activity (Khan et al., 2013; Berli et al., 2016). In terms of recipients’ well-being, however, findings of actual support receipt are inconsistent (for an overview see Rafaeli and Gleason, 2009). Overall, most focus of the social support literature has been on outcomes in support recipients. The phenomenon that providing support may entail benefits for providers is less understood. This study aims to contribute to evidence on the effects of providing social support in daily life on health behavior and emotional and relational well-being using a dyadic approach with romantic overweight couples intending to increase their physical activity.

### Social Support and Health

Social support has been defined as “social resources that persons perceive to be available or that are actually provided to them by nonprofessionals” (Cohen et al., 2001). Social support can be conceptualized as retrospective reports of supportive interactions in the past, reported by either recipients (i.e., *received* support) or by providers (i.e., *provided* support) (Schwarzer and Knoll, 2007). Importantly, this has to be distinguished from *perceived* support that refers to a prospective assessment of help perceived as available should need arise (e.g., Uchino, 2009). Different functions of support include providing comfort or listening (i.e., emotional support) or providing material, practical help (i.e., instrumental support) (Schwarzer and Knoll, 2007). Receiving support has been proposed to result in, among others, improved emotional (e.g., negative and positive emotions), relational (e.g., trust, closeness, feeling valued, and respected), and behavioral (e.g., health and lifestyle behaviors) outcomes (Feeney and Collins, 2015). However, benefits may not be limited to the individual receiving the support. Providing support to one’s romantic partner may even be more important for one’s health than receiving it (e.g., Knoll et al., 2007).

### Benefits for Support Providers

Compelling evidence exists that older adults who provided higher levels of support to others had a reduced risk for mortality 5 years later (Brown et al., 2003) and lower morbidity (Brown et al., 2005), independent of levels of received support. McClellan et al. (1993) had already demonstrated that among dialysis patients with end-stage renal disease, levels of giving support to family and friends were higher in those who survived than those who died 1 year later. Trait support provision was moreover associated with cardiovascular health (e.g., lower ambulatory blood pressure; Piferi and Lawler, 2006). Using an experimental design, Inagaki and Eisenberger (2016) could show that providing support to a friend (i.e., writing a supporting note vs. writing about route to school/work) prior to a stressful experience influenced the physiological stress response by reducing systolic blood pressure but did not have an effect on self-reported psychological stress or salivary cortisol levels.

Moreover, providing support to others has shown to be associated with better mental health in providers, including decreased depressive symptoms in individuals mourning for a spouse (Brown et al., 2008) and decreased symptoms of depression and anxiety in college students (e.g., Crocker et al., 2010). In couples undergoing in vitro fertilization, spousal provision of support was associated with a decrease in own negative affect and increase in own positive affect (Knoll et al., 2007), suggesting that support provision may bolster the provider’s feelings of well-being. The authors assume that providing support to someone else should increase self-esteem and well-being because it makes the provider feel needed, important, and valuable (esteem enhancement; Batson and Powell, 2003). Providing support may further increase the sense of reciprocity within the couple (Ryon and Gleason, 2018). Other mechanisms discussed to explain these positive effects include that providing support may also distract from and facilitate reappraisal of own problems, make providers feel more energized, and efficacious, and strengthen networks (Crocker et al., 2017; Liu et al., 2020).

Providing support also seems important for the development and maintenance of relationships, by cultivating satisfying, trusting, and intimate relationships (Feeney and Collins, 2015). According to Cutrona (1996), social support should be linked with more positive aspects and less negative aspects of relationship quality via reducing conflicts and preventing emotional withdrawal, reducing the risk for depression, and increasing intimacy. Correlational evidence shows that reports of providing support (e.g., Brunstein et al., 1996) as well as observer-rated support provision during couple conversations (Lawrence et al., 2008; Jensen et al., 2013) were positively associated with relationship outcomes in providers, particularly among men. Among newly married couples, wife’s support provision during a discussion about a personal stressor predicted relationship satisfaction and distress 2 years later (Pasch and Bradbury, 1998). Prospective positive associations were also found in men receiving radical prostatectomy (Knoll et al., 2009): Patients’ accounts of support provision to their partner prior to the operation significantly predicted their relationship satisfaction 1 year after surgery, even after controlling for the patient’s accounts of support receipt from their partner, and presurgery levels of relationship satisfaction.

It is important to note, however, that providing support can also be costly. It is well documented that caregiver burden is related to lower indicators of physical health, lower intimacy and relationship quality, and increased stress (see Adelman et al., 2014; Crocker et al., 2017). In particular, providing care for a close other with a chronic condition can be burdensome and limit personal resources (Crocker et al., 2017; Liu et al., 2020). Caregivers may experience multiple stressors including the strain of patients’ disabilities, exposure to their suffering, and restricted personal and social life. The burden of caregiving tends to be greater the closer the caregiver is (Rafaeli and Gleason, 2009). However, some studies also extended the positive findings of support provision to the caregiving context. For example, Brown et al. (2009) found that individuals who provided a high number of hours of care to their spouse had lower mortality rates. According to the authors, the perception of the patient’s suffering may indeed be harmful, but the caregiver’s compassion could still be beneficial for his or her outcomes.

### Support Provision in Daily Life

Overall, research indicates that individuals who provide more support to others such as the romantic partner seem to display better physical, emotional, and relational well-being. This approach, focusing on differences in support provision *between* individuals, can answer the question of whether a trait disposition of giving support to others relates to better health. Using a daily diary design can address the question of whether the process of providing support to others in daily life is, *within* persons, associated with health benefits relatively close in time. Gleason et al. (2003) for example found that providing support to the partner one day was associated with less negative mood that same day. Similarly, positive effects on own same-day mood or well-being indicators were found in patients with multiple sclerosis and their partners (Kleiboer et al., 2006), in cancer patients and caregivers following stem cell transplantation (Kroemeke et al., 2019), same-gender undergraduate friend dyads (Morelli et al., 2015), and spouses of individuals with military posttraumatic stress (Carter et al., 2019). Positive effects of daily reports of providing support to the romantic partner on providers’ relationship outcomes were also confirmed, e.g., higher daily intimacy in couples coping with breast cancer (Belcher et al., 2011), more feelings of closeness and decreased negative affect in examinees preparing for the bar exam (Gleason et al., 2008), or higher daily relationship satisfaction, but only in the context of positive event disclosure (Gosnell and Gable, 2015). These associations with emotional and relational well-being have, however, so far not been tested in the context of health behavior change.

### Support Provision and Health Behavior

What has been less discussed in the literature as a potential explanation for the effects of support provision on physical health is a health behavior path. Providing support might not only impact on health by promoting health behaviors in recipients (cf. Berkman et al., 2000; Feeney and Collins, 2015) but also promote engagement in health behaviors in providers themselves, for example due to strengthened self-regulation. More specifically, providing support to a close other in daily life (e.g., encouraging, reminding of goals, providing information, or appropriate materials) is highly likely to activate goals and trigger self-regulatory strategies (self-monitoring or planning) in providers themselves and help them pursue their own health goals.

So far, a few studies exist that investigated the effects of providing social support on health behavior in the context of substance use, with mixed results. Giving help to other juveniles, as opposed to receiving help, significantly reduced the risk of relapse in alcohol and other drug use during 12 months following an addiction treatment (Johnson et al., 2018). A main effect of providing support through online social support groups on alcohol and drug use 6 and 12 months later was, however, not confirmed (Liu et al., 2020). Using a dyadic approach with dual-smoker couples, Lüscher and Scholz (2017) did not find evidence that female partners who reported providing smoking-specific support to their male partners a month after quitting jointly were more likely to be abstinent. However, zooming in on a daily perspective based on dyadic daily diary data from the same dual-smoker couples attempting to quit smoking jointly (Lüscher et al., 2017), the authors could show that on days when men and women reported providing more support to their partner than usual (within-person fluctuations in support provision), men and women also reported smoking less cigarettes that day. To the best of our knowledge, no studies have tested the effects of support provision in other health behavior contexts, such as physical activity, and focused comprehensively on provider’s own health behavior and emotional and relational well-being.

#### The Present Study

**FIGURE 1:**
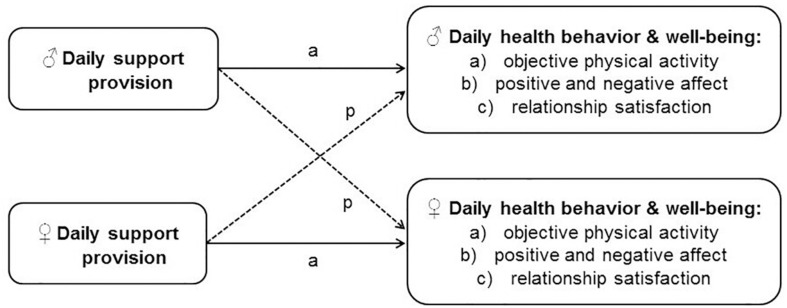
Conceptual model based on the Actor–Partner Interdependence Model (APIM). The model depicts the male (♂) and female (**♀**) couple members and their reports of the predictor and outcome variables. Continuous lines symbolize the actor effect (a), and dashed lines symbolize the partner effect (p). Separate analyses were conducted for each outcome.

To summarize, evidence suggests that persons who provide support to a close other also show better health. Existing diary work also suggests that within persons, providing support to another person in daily life is associated with improved own daily emotional and relational well-being. However, so far these associations have not been investigated in support contexts such as pursuing health behavior change. Evidence on the effects of support provision on the provider’s own health behavior is mixed and limited to the context of substance use. The aim of the present study is to comprehensively examine the effects of daily support provision on the provider’s own health behavior and emotional and relational well-being in romantic overweight couples striving to increase their physical activity in everyday life. To capture the dynamic process of support provision and its relatively short-term effects, we strictly focus on within-person associations, taking between-person means into account. As can be seen in Figure 1, we hypothesized that higher daily support provision relates to (a) higher own objective physical activity, (b) higher own positive and lower own negative affect, and (c) higher own relationship satisfaction that same day in male partners and female partners (actor effects “a”). Using the framework of the Actor–Partner Interdependence Model (APIM; Kenny et al., 2006) with reports from both partners will allow to disentangle the effects of one’s own and one’s partner’s support provision on the outcomes.

## Materials and Methods

The present study is a secondary analysis of data collected at the 6-month follow-up of an intervention study to promote daily physical activity in inactive, overweight, or obese couples intending to become physically active (“DYACTIC;” for details please see Scholz and Berli, 2014). The single-blind randomized controlled trial (ISRCTN15705531) was funded by the Swiss National Science Foundation (PP00P1_133632/1) and approved by the Internal Review Board of the University of Bern, Switzerland (2011-12-36206). In brief, the intervention consisted of an information leaflet with physical activity recommendations at the time of the study (engaging in 30 min or more of at least moderate activity every day, performed in bouts of at least 10 min; BASPO, 2009) for all participants, a goal-setting task, and action control text messages delivered across an intervention period of 14 days. For detailed information on the recruitment, sampling procedure, intervention, and intervention effects of the trial, please see Berli et al. (2016). Below is a concise description of the procedures and measures uniquely relevant for the present study.

### Design and Participants

Participants were heterosexual adult couples living in a committed relationship for at least 1 year and cohabitating for at least 6 months. Both partners were overweight or obese (body mass index [BMI] ≥25 kg/m^2^), did not meet physical activity recommendations (BASPO, 2009), but intended to increase their physical activity levels. Eligible couples were recruited from the community via flyers, advertisements, and a market research institution. They were invited to the lab where they provided written informed consent and completed an online questionnaire and were randomized to an intervention group (*n* = 61) or a control group (*n* = 62). After baseline, they completed a 28-day diary period with electronic end-of-day diaries and assessment of physical activity via an accelerometer (14 days of intervention, 14 days of assessments only). One and six months after baseline, they returned to the lab for a follow-up assessment. Following the 6-month follow-up assessment, they completed another 14-day diary period with assessments only. This follow-up diary period provides the basis for the secondary analysis of the present study. For the 14 consecutive days, couple members were instructed to independently fill in electronic end-of-day diaries within 1 h of going to bed. They were asked not to discuss their answers with their partners. Additionally, they were asked to wear an accelerometer to objectively assess daily physical activity. At the end of this period, they returned the devices via mail. Couples completing the study were compensated with a total of CHF 200 (=approximately 114 USD).

Of the 121 couples participating at baseline, 99 couples (82%) completed the follow-up diary assessments and comprised the final sample for this study. On average, couples had been living in a committed relationship for 19.12 years (*SD* = 14.31) and cohabitating for 17.30 years (*SD* = 14.39). 69.7% were married, and 56.6% had children with their partner. On average, women were 45.31 years old (*SD* = 13.51, range: 23–72); men were 47.29 years old (*SD* = 13.94, range: 22–75). The average BMI was for women 30.87 (*SD* = 4.94, range: 25–50) and for men 31.30 (*SD* = 4.98, range: 25–62). Due to technical issues, two couples did not provide any accelerometer data, which resulted in a sample of 97 couples for the analysis of daily physical activity.

### Measures

Across the 14 consecutive days of the follow-up diary, every evening both partners reported on their daily support provision and emotional and relational well-being, with high overall completion rates (*n* = 2630 [94.9%] of 2772 possible diary days). All items were administered in German. The item examples below have been translated into English. Table 1 gives an overview of the descriptive statistics of the main variables. For affect and social support, we further calculated two reliability estimates (Cranford et al., 2006): A between-person reliability *R*_*kf*_, which indicates whether someone tends to be high or low on a given scale, and a within-person reliability *R*_*c*_, which indicates the reliability of measuring systematic change in ratings over time across individuals.

**TABLE 1:** Available data, descriptive statistics, and correlations between variables of interest.

	*N*	n	M_*B*_	SD_*B*_	SD_*W*_	Range	ICC	1	2	3	4	5
1. Support provision	99 (198)	2630	1.37	0.92	0.82	0 – 4	0.47	−	0.13**	−0.12**	0.25**	0.15**
2. Positive affect	99 (198)	2630	0.57	0.14	0.11	0.12 – 0.93	0.56	0.08	−	−0.33**	0.23**	0.11**
3. Negative affect	99 (198)	2630	0.17	0.13	0.09	0.00 – 0.59	0.60	0.28**	−0.31**	–	−0.26**	−0.08**
4. Relationship satisfaction	99 (198)	2630	0.72	0.17	0.13	0.22 – 1.00	0.55	–0.03	0.33**	−0.33**	–	0.08**
5. MVPA (in minutes)	97 (194)	2428	48.26	24.40	27.02	1.25 – 129.43	0.37	–0.03	0.02	–0.06	0.05	–
6. Age (years)								0.24**	0.03	–0.11	–0.09	−0.30**
7. Relationship length (years)								0.14^†^	0.03	–0.08	−0.21**	−0.21**
8. Body mass index (kg/m^2^)								–0.07	–0.08	0.01	0.06	−0.15*
9. Kids (no = 0; yes = 1)								–0.04	–0.02	–0.01	−0.19**	–0.02

N = number of couples (individuals); n = number of available diary days; M_B_ and SD_B_ show the mean and standard deviation of person-specific mean levels (between-person level); SD_W_ = average within-person standard deviation (within-person level); ICC = intra-class correlation (variance due to stable between-person variability). Between-person correlations for variables 1 through 9 are shown below diagonal. Within-person correlations for variables 1 through 5 are shown above diagonal. ^†^p < 0.10, *p < 0.05 **p < 0.01.

#### Daily Support Provision

Both partners indicated the extent to which they provided activity-specific social support to the other partner that day, with one item each on emotional and practical support (adapted from Bolger et al., 2000): “Today, I provided *emotional* [or: *practical*] support to my partner in terms of his/her physical activity.” The response format was 0 (today not at all true) to five (today completely true). Before answering the items, participants were presented with a short description and some examples of emotional (e.g., comfort or encouragement) and practical (e.g., advice or information) support. A mean score of support provision was calculated due to high correlation of emotional and practical support (between: *r* = 0.93, *p* < 0.001; within: *r* = 0.70, *p* < 0.001). Reliability scores were *R*_*kf*_ = 0.99 and *R*_*c*_ = 0.83.

#### Daily Positive and Negative Affect

Both partners were asked to rate their affect during that day, using the short form of the Positive and Negative Affect Schedule (Thompson, 2007) with five items each. Example items are “Today I feel excited” for positive affect and “Today I feel distressed.” The response format was 0 “today not at all true” to 5 “today completely true.” To facilitate interpretation of results and comparability between the outcome variables, positive and negative affect was rescaled to a 0 to 1 scale (0 = 0, 1 = 0.2, 2 = 0.4, …, 5 = 1, etc.). Reliability scores were *R*_*kf*_ = 0.99 and *R*_*c*_ = 0.75 for positive affect; *R_*kf*_* = 0.99 and *R*_*c*_ = 0.70 for negative affect.

#### Daily Relationship Satisfaction

Both partners indicated the extent to which they were satisfied with their relationship that day, using the following item adapted from the DAS-7 (Hunsley et al., 2001): “How did you experience your relationship today?”. The response format was 0 “Today terrible,” 3 “Today ok,” to 6 “Today wonderful.” To facilitate interpretation of results and comparability between the outcome variables, relationship satisfaction was rescaled to a 0 to 1 scale (0 = 0, 1 = 0.17, 2 = 0.33, 3 = 0.5, 4 = 0.67, 5 = 0.83, 6 = 1).

#### Daily MVPA (in Minutes)

GT3X+ monitors (ActiGraph, Pensacola, FL, United States) worn at the hip during waking hours were used to assess both partners’ daily moderate-to-vigorous physical activity (MVPA). The GT3X+ measures acceleration on three axes (providing a composite measure, i.e., “vector magnitude”) and is a reliable and valid instrument for measuring physical activity levels (Sasaki et al., 2011). For each participant, the total amount of minutes per day that was spent in at least moderate or vigorous physical activity (>2690 cpm in vector magnitude; Sasaki et al., 2011) was calculated. Non-wear time was filtered and eliminated from further analysis based on an algorithm of ≥ 90 min of consecutive zeros in vector magnitude (Choi et al., 2011). Only days with at least 10 h of wear time were included in the analyses. This resulted in *n* = 2,428 [89.4%] available diary days of 2,716 possible diary days across the 97 couples and served as basis for the analysis of physical activity. For more details on data processing, see Berli et al. (2016).

### Data Analysis

Data from the 99 male and female partners (=198 individuals) were analyzed using the Actor–Partner Interdependence Model (APIM; Kenny et al., 2006). Actor (the individual) and partner (the individual’s partner) reports of daily support provision were used, allowing to estimate the extent to which the outcome is related to one’s own and one’s partner’s predictor scores while controlling simultaneously for the effect of both. To account for the nested data structure with repeated measures among male and female couple members, we employed multilevel modeling using a two-level statistical model for distinguishable dyads (Bolger and Laurenceau, 2013). Male and female partners’ reports of support provision were first decomposed into individual mean levels across the diary days (i.e., between-person variance) and the daily fluctuations around these means (i.e., within-person variance). The between-person predictor variables were grand-mean centered to allow for a meaningful interpretation of the intercept. This allowed us to analyze whether daily fluctuations from an individual’s typical (average) level of support provision were associated with the outcomes (within-person association), while controlling for the individual’s mean level.

We modeled couple members’ outcomes on a given day as a function of their own fluctuation in support provision and their partner’s fluctuation in support provision that same day (within-person actor and partner predictors), adjusting for the mean level of own and partner’s support provision (between-person actor and partner predictors). For model parsimony, we constrained actor and partner effects to be equal across gender. Sensitivity analyses revealed no differences between male and female partners in these effects. We, however, added gender as a covariate and adjusted for linear time trends using a linear time variable centered on the first diary day (day 1 = 0, day 2 = 1, … day 14 = 13). To rule out confounders of the within-person associations, we included a dummy variable weekdays (= 0) vs. weekends (= 1) in all analyses. In the analysis predicting physical activity, we moreover included hours of device wear time (centered around the grand mean) as a covariate to adjust for the potential impact of varying levels of accelerometer wear time. In all analyses, we specified a maximal random-effects structure (Barr et al., 2013) including random intercept and slopes for all lower-level predictors, using a variance component (VC) covariance structure^1^. In case of non-convergence, the random-effects structure was successively reduced, eliminating effects with insufficient random variance until convergence was met. For each outcome of interest, we ran a separate linear mixed model using IBM SPSS version 26.

We ran a set of sensitivity analyses [see Supplementary Tables 1–3] to test whether results hold when including (1) both partner’s reports of received support, (2) reports of daily time spent together, and (3) intervention group^2^ as well as socio-demographic variables that showed to be associated with the respective outcome as covariates in the analysis. In none of the models, results changed, so we reported the more parsimonious models below.

## Results

Table 1 displays the descriptive statistics and bivariate associations among the main variables. Intraclass correlations, a measure of the degree of dependence of data points (Kreft and DeLeeuw, 1998), ranging between 0.37 and 0.60 indicate that roughly between one and two thirds of the total variance was due to stable between-person differences. At the within-person level, all outcome variables were significantly associated with each other, with small to moderate negative correlations for daily negative affect and positive correlations for positive affect, relationship satisfaction, and MVPA. At the between-person level, a higher level of positive affect across the 14 days was moderately associated with a lower level of negative affect and higher level of relationship satisfaction, and a higher level of negative affect was moderately associated with a lower level of relationship satisfaction. MVPA was not inter-correlated with the other outcomes.

Using linear mixed models, we tested the assumption that higher daily support provision would relate to male and female partners’ (a) higher own MVPA, (b) higher own positive affect and lower own negative affect, and (c) higher own relationship satisfaction that same day (actor effects). For a complete overview of the results, please see Table 2.

**TABLE 2:** Parameter estimates from mixed models testing the effect of support provision on couples’ daily MVPA, positive and negative affect, and relationship satisfaction.

	MVPA (in minutes)		Positive affect		Negative affect		Relationship satisfaction	
Fixed effects	Estimate	SE	Estimate	SE	Estimate	SE	Estimate	SE
Intercept	48.69***	2.41	0.58***	0.01	0.18***	0.01	0.70***	0.01
Gender	3.63	2.45	–0.02	0.02	−0.03^†^	0.02	–0.02	0.02
Time	0.10	0.15	−0.002^†^	0.001	0.001	0.001	0.002	0.001
Weekend	–2.61	2.06	0.01	0.01	−0.03***	0.01	0.05***	0.01
Wear time (in hours)	2.32***	0.40	−	−	−	−	−	−
*WITHIN effects*								
Own support provision (actor effect)	3.81***	0.94	0.02***	0.003	−0.01**	0.003	0.03***	0.004
Partner’s support provision (partner effect)	4.55***	0.72	0.002	0.003	–0.003	0.003	0.01***	0.004
*BETWEEN effects*								
Own support provision (actor effect)	–1.18	1.88	0.01	0.01	0.04***	0.01	–0.004	0.01
Partner’s support provision (partner effect)	2.56	1.87	–0.01	0.01	–0.02	0.01	0.005	0.01
**Random effects (variances)**								

**Level 2 (between-person)**								
Intercept	418.85***	67.95	0.01***	0.002	0.01***	0.001	0.02***	0.003
Gender	425.99***	83.27	0.03***	0.004	0.02***	0.003	0.03***	0.004
Time	0.05	0.27	< 0.001***	<0.001	< 0.001^†^	<0.001	<0.001	<0.001
Weekend	201.31***	56.76	0.001	0.001	0.001^†^	<0.001	0.002**	0.001
Wear time (in hours)	1.47	2.15	−	−	−	−	−	−
Own support provision (actor effect)	24.93**	9.43	<0.001	<0.001	<0.001	<0.001	0.0004*	<0.001
Partner’s support provision (partner effect)	−^*a*^	−^*a*^	<0.001	<0.001	<0.001	<0.001	<0.001	<0.001
**Level 1 (within-person)**								
Residual	746.44***	25.49	0.01***	<0.001	0.01***	<0.001	0.02***	0.001
Autocorrelation	0.004	0.03	0.22***	0.02	0.19***	0.03	0.20***	0.03

For model on MVPA, N = 97 (194) couples (individuals) with a maximum of 28 days, n = 2259 available days; for models on positive and negative affect and relationship satisfaction, N = 99 (198) couples (individuals) with a maximum of 28 days, n = 2181 available days. SE = standard error. Gender is coded as female = −0.5 and male = 0.5. ^a^Due to non-convergence, not all random effects could be computed. ^†^p < 0.10, *p < 0.05, **p < 0.01, ***p < 0.001.

### Daily Moderate-to-Vigorous Physical Activity

As indicated by the intercept (i.e., when all covariates equal zero), participants’ average level of MVPA on the first diary day was 48.7 min. Male and female partners did not differ in their MVPA levels (*b* = 3.63, *p* = 0.143). MVPA did not significantly change over time (*b* = 0.10, *p* = 0.506) and was not different on weekend days versus weekdays (*b* = −2.61, *p* = 0.206), providing more support than usual (one unit above the person-specific mean) to the other partner on a given day predicted higher own MVPA (*b* = 3.81 min, *p* < 0.001) that same day. This is in line with Hypothesis 1a on actor effects.

In addition, we found that providing more support than usual on a given day also predicted higher MVPA in the other partner (*b* = 4.55 min, *p* < 0.001) that same day (partner effect). There was considerable variation between individuals in their average level of MVPA (random intercept), and the extent to which own support provision related to own MVPA (random slope for actor effect): The corresponding SD of 4.99 (=√24.93) for the random slope of provided support indicates that 95% of the population varies between ± 9.79 min (=1.96 × 4.99) of the average effect.

### Positive and Negative Affect

As indicated by the intercept (i.e., when all covariates equal zero), the average level of *positive affect* on the first diary day was 0.58 (on a scale from 0 to 1) and male and female partners did not differ in these initial levels (*b* = −0.02, *p* = 0.220). Positive affect did not significantly change over the diary days (*b* = −0.002, *p* = 0.081) and was not different on weekend days vs. weekdays (*b* = 0.01, *p* = 0.245). In line with hypothesis 1b on actor effects, providing more support than usual (one unit above the person-specific mean) to the other partner on a given day, predicted higher own positive affect that same day (*b* = 0.02, *p* < 0.001).

In addition, providing more support than usual on a given day did not predict positive affect in partners (*b* = 0.002, *p* = 0.574) that same day (partner effect). Moreover, there was considerable variation between individuals in their average level of positive affect (random intercept), but not the extent to which own support provision related to own positive affect (random slope for actor effect).

The average level of *negative affect* on the first diary day was 0.18 (on a scale from 0 to 1), and male and female partners did not differ in these initial levels (*b* = −0.03, *p* = 0.066). No significant change over the diary days emerged for negative affect (*b* = 0.001, *p* = 0.381). On weekends, negative affect was lower compared to weekdays (*b* = −0.03, *p* < 0.001). In line with results on positive affect and Hypothesis 1b on actor effects, providing more support than usual (one unit above the person-specific mean) to the other partner on a given day predicted lower own negative affect that same day (*b* = −0.01, *p* < 0.01).

In addition, providing more support than usual on a given day did not predict negative affect in partners (*b* = –0.003, *p* = 0.197) that same day (partner effect). Again, there was considerable variation between individuals in their average level of negative affect (random intercept), but not the extent to which one’s own support provision related to own negative affect (random slope for actor effect).

### Daily Relationship Satisfaction

The average level of *relationship satisfaction* on the first diary day was 0.70 (on a scale from 0 to 1) and male and female partners did not differ in these initial levels (*b* = −0.02, *p* = 0.170). Relationship satisfaction did not significantly change over time (*b* = 0.002, *p* = 0.101) but was higher on weekend days compared to weekdays (*b* = 0.05, *p* < 0.001). In line with Hypothesis 1c on actor effects, providing more support than usual (one unit above the person-specific mean) to the other partner on a given day predicted higher own relationship satisfaction that same day (*b* = 0.03, *p* < 0.001).

Additionally, providing more support than usual on a given day also predicted higher relationship satisfaction in partners (*b* = 0.01, *p* < 0.001) that same day (partner effect). Moreover, there was considerable variation between individuals in their average level of relationship satisfaction (random intercept), and the extent to which own support provision related to own relationship satisfaction (random slope for actor effect): The corresponding SD of 0.02 (=√0.0004) for the random slope of provided support indicates that 95% of the population varies between ±0.04 (=1.96 × 0.02) of the average effect.

### Sensitivity Analyses

Importantly, sensitivity analyses revealed that for all outcomes of interest the hypothesized actor effects remained significant when adjusting for both partners’ reports of *received* support (see Supplementary Table 1). This indicates that the within-person effects of providing support for providers are independent of potential effects of receiving support from the partner. Moreover, results did not change when adjusting for daily time spent together (see Supplementary Table 2) or socio-demographic variables and intervention group (see Supplementary Table 3).

## Discussion

This study was designed to examine whether providing daily support to the romantic partner in the context of pursuing physical activity goals would be associated with better health behavior and well-being in providers. Using a dyadic approach with overweight and inactive romantic couples, we particularly examined the effects of support provision on a comprehensive set of health behavior and emotional and relational well-being, including an objective assessment of physical activity behavior via accelerometers.

In line with our hypotheses, we found that higher daily support provision was associated with (a) higher own objective MVPA levels, (b) higher own positive affect and lower own negative affect, and (c) higher own relationship satisfaction that same day in men and women (actor effects). These effects were independent of the effect of the other partner’s report of support provision (partner effects). Although not the focus of the present paper, partner effects of support provision were found for MVPA, as documented in previous studies (Berli et al., 2018a; e.g., Khan et al., 2013), and relationship satisfaction. No associations between partner reports of support provision and own positive or negative affect emerged, reflecting the rather inconsistent empirical evidence on support and recipient’s well-being (Rafaeli and Gleason, 2009).

Overall, the results on actor effects are in line with previous findings on benefits of support provision for providers’ physical health (e.g., Brown et al., 2003, 2005) and mental well-being (e.g., Knoll et al., 2007; Brown et al., 2008). They extend current findings by demonstrating that providing support is associated with better health behavior in providers. More specifically, participants engaged in almost four more minutes of MVPA, respectively, on days they reported providing more activity-specific support to their partners than usual. Given that differences in support provision across days could be up to five units (with a response format of 0 to 5) and physical activity was objectively measured using accelerometers, this effect is quite substantial. It is also comparable to the effect of support provision on recipients’ MVPA (around five more minutes of MVPA on days their partners reported providing more support than usual). Possible mechanisms for the effect of providing support could include jointly engaging in activity behaviors (cf. Berli et al., 2018a), increased own self-regulation (e.g., intentions, planning or monitoring), and self-efficacy, possibly via vicarious experience (e.g., the successful partner serves as role model). Previous research has shown that couples’ health behavior change (Jackson et al., 2015) as well as self-regulation processes in daily life (Berli et al., 2018b) are highly linked. Social support could be one mechanism that contributes to this link (e.g., reminding you to do x also reminds me to do x). The proposed mechanisms may be particularly likely in the present sample where both partners were overweight and committed to engaging in regular activity. Thus, such robust findings might not generalize to more asymmetrical couple constellations, e.g., when only one partner is overweight and intending to achieve the recommended physical activity levels. The effect on providers’ own physical activity behavior is in line with findings from a previous diary study in the context of smoking cessation, demonstrating that in both male and female romantic partners, providing more smoking-specific support on a given day related to less self-reported cigarettes smoked that day (Lüscher et al., 2017). Other studies investigating this association in the context of alcohol and substance use with a between-person focus (Lüscher and Scholz, 2017), across larger time intervals as well as outside the romantic relationship (Johnson et al., 2018; e.g., Liu et al., 2020) resulted in rather mixed evidence.

Together, these results suggest that health behaviors may, apart from physiological processes (e.g., Piferi and Lawler, 2006; Inagaki and Eisenberger, 2016), provide an alternate pathway through which support provision impacts on providers’ long-term health outcomes. While health behaviors have been generally acknowledged as one potential pathway from social networks, and more specifically social support, to health (e.g., Berkman et al., 2000), this pathway has been neither explicitly proposed nor tested as being carried, at least in part, also by the support providers. Future studies should test this assumption in the context of other health behaviors.

Moreover, results suggest that providing health-related social support to the romantic partner is also related with providers’ higher emotional and relational well-being, supporting and extending previous diary work beyond the context of stress (e.g., Gleason et al., 2008; Carter et al., 2019) or illness (e.g., Kleiboer et al., 2006; Belcher et al., 2011; Kroemeke et al., 2019). One explanation may again be that supporting the partner for example by engaging in activity together may foster feelings of companionship and cohesion. Companionship (e.g., participating in shared leisure activities) has also been associated with better psychological and relational well-being independently from social support (Rook, 2015). Findings based on end-of-day diaries suggest that instances of providing support in daily life are relatively closely linked with daily better mood and higher daily relationship satisfaction. This offers a complementary view to the relationship enhancement model of social support (Cutrona et al., 2005), assuming that perceiving the partner as a consistent and reliable source of support determines relationship quality and stability via increased trust.

As mentioned previously, providing support or care for a close other may not always have positive effects, particularly in the context of chronic conditions. In contrast to caregiving, however, the context of health behavior change does not require providers to provide constant support or physically demanding care. Instead, the decision to provide support can generally be freely made and is thus assumed to be of low cost. According to Inagaki and Orehek (2017), beneficial outcomes of support provision seem to depend on two factors: Whether or not an individual can freely choose to provide support, and whether or not an individual thinks his or her support is effective. Indeed, research has shown that autonomous motivation to help yields benefits for the helper and recipient (e.g., Weinstein and Ryan, 2010). Sensitivity analyses also did not reveal that the effect of daily support provision on well-being was less advantageous for individuals with high overall levels of support compared to individuals with lower overall levels of support. More systematic research should be devoted to the question of when, why, and how giving support is beneficial (cf. Inagaki and Orehek, 2017).

It is also likely that the close relationship context is a rather favorable context for positive outcomes in support providers. Romantic partners, in particular, are at the source of receiving and providing support to each other in daily life, when it is most needed. Due to their shared history, they might be able to provide support responsively. This not only may be linked with more effective support outcomes in recipients but also may make it more likely for providers to feel efficacious, satisfied, and valuable—aspects that have been theorized to explain increased well-being in providers (cf. Knoll et al., 2007; Liu et al., 2020). Yet, the considerable variation in the effect of providing support on own physical activity and relationship satisfaction across individuals suggests that the provider effect is not uniformly strong for everyone and may even be negative. Potential explanations may include characteristics of the relationship (e.g., overall relationship quality, equity), of the individual (e.g., altruistic motives, goal motivation), or of the support interaction (e.g., support reciprocity; Ryon and Gleason, 2018).

Also, type of support, e.g., whether support is overt and visible or covert and invisible (Bolger et al., 2000), could play an important role in determining positive provider effects. For example, it has been shown that invisible support was associated with decreased mood in support providers, particularly when perceived relationship quality was low (e.g., König et al., 2016). This effect can potentially be explained by a lack of acknowledgment of providers’ efforts to support the recipient. In contrast, research on support recipients has shown that visible support can be costly for recipients’ mood while invisible support appears to avoid such costs, protecting recipients’ self-efficacy (e.g., Girme et al., 2018). Under which conditions which type of support (visible vs. invisible) is associated with most positive outcomes in support providers, however, needs further examination. This could be a first step toward a better understanding of the relational, individual, and contextual boundary conditions of effective support provision.

### Strengths and Limitations

This study has several strengths. To shed light on the daily processes of support provision in romantic couples’ everyday life, we collected daily reports from both male and female partners on support and a broad range of health outcomes. Moreover, applying the APIM framework allowed us to disentangle the effects of one’s own and one’s partner’s support provision on both persons’ outcomes. With this, the effect of own support provision on one’s own daily health cannot be attributed to the effect that the other partner’s support provision may have had on one’s own health. Moreover, using an objective measure of health behavior to assess daily physical health is a particular advantage that can produce reliable findings by avoiding shared measurement variance. While potential recall bias of self-report measures should have been reduced due to the diary setting, the issue of shared measurement variance remains with the use of self-report measures for support provision and emotional and relational well-being. An objective assessment of support provision would be ideal. While observations in the lab have been conducted in previous studies on support provision and relationship health (e.g., Lawrence et al., 2008; Jensen et al., 2013), a future way to go could lie in naturalistic observations of support instances in daily life via audio recordings (cf. Lüscher et al., 2019), using an electronically activated recorder (EAR; Mehl et al., 2001).

Despite the many benefits of intensive longitudinal data, it is important to note that causality cannot be established. The predictive direction might also be the other way around. Particularly, feeling happier might facilitate support behaviors. Being in a good mood has previously shown to be associated with an increased likelihood to provide support (Iida et al., 2008). Being happy with the partner, due to moments of intimacy, could also enhance the probability to provide support. Previous research has shown that individuals who are satisfied with their relationship expect and perceive their spouses to be more supportive (cf. Frazier et al., 2003); however, reciprocal associations are highly likely. Importantly, in sensitivity analyses we could rule out the possibility that the associations are simply due to spending more time together.

Relatedly, the present data do not allow to detect potential sequential processes of the health outcomes, e.g., that behavioral goal achievement elicits subsequent feelings of well-being or that relationship satisfaction fosters better subsequent mood. Previous research for example indicated that spousal support receipt predicted higher goal progress which predicted increased positive affect and relationship quality and decreased physical symptoms the following day (Jakubiak and Feeney, 2016). However, the authors also noted that a reverse pattern of well-being predicting subsequent goal progress was also supported by the data, suggesting that bidirectional associations are plausible. The relationship enhancement model of support (Cutrona et al., 2005) moreover proposes that relationship satisfaction leads to better physical and mental health in the long run. Whether such temporal dynamics also unfold at a micro-time level (e.g., from day-to-day) within persons is, however, unclear and needs further investigation. Unfortunately, the present data are not suitable to test such assumptions. More fine-grained assessments (e.g., several within-day assessments) could help to establish a predictive order. However, ideally, experimental designs are warranted.

### Implications and Conclusion

The present findings highlight the importance of health-related support interactions in close relationships for providers’ health behavior and emotional and relational well-being in daily life. Theoretical frameworks have identified several pathways (e.g., behavioral, physiological, and psychological) through which support impacts on well-being indicators and long-term health (e.g., Berkman et al., 2000; Feeney and Collins, 2015), with a more or less explicit focus on support recipients. Given the accumulating evidence on benefits of support provision, frameworks should more explicitly acknowledge how such pathways flow through support providers.

With evidence accumulating on independent effect of support provision on own health outcomes, implications for intervention work emerge. Apart from interventions that aim to help people feel supported or that are designed to instruct spouses to support the person in need, new interventions need to be developed. This could for example involve to identify possibilities to provide support to others. Dyadic interventions which involve both members of a dyad, and have increasingly been used to improve health (cf. Scholz et al., 2020), might be particularly suitable and could maximize intervention effectiveness.

In sum, shifting the focus away from the support recipient to examine outcomes in support providers is of particular relevance. Providing support in daily life to a close other pursuing health goals seems to be associated with benefits for providers’ health in terms of their health behavior, emotional well-being, and relationship.

## Data Availability Statement

The raw data supporting the conclusions of this article will be made available by the authors, without undue reservation, to any qualified researcher.

## Ethics Statement

The studies involving human participants were reviewed and approved by the Internal Review Board of the Faculty of Arts and Social Sciences of the University of Bern, Switzerland. The patients/participants provided their written informed consent to participate in this study.

## Author Contributions

US acquisited funding and designed the empirical study. CB supervised data collection and led the project administration, conceptualized the research goals and aims of the present manuscript. CB analyzed the data and wrote the original draft of the manuscript. US and PS critically reviewed and edited the manuscript. All authors approved the final submitted version of the manuscript.

## Conflict of Interest

The authors declare that the research was conducted in the absence of any commercial or financial relationships that could be construed as a potential conflict of interest.

## Footnotes

1 Because a full random-effects variance–covariance structure (using an unstructured matrix) did not converge, we simplified to a more parsimonious variance component (VC) covariance structure on the random effects, where we could estimate the variances, but set the covariance between the random effects to zero.

2 Preliminary analyses did not reveal any significant differences between participants of the intervention and control group in terms of their mean support provision, positive and negative affect, relationship satisfaction, and MVPA across the 14 diary days (p’s all >0.05).
