# Supplementary material for: “We Feel Good”: Daily Support Provision, Health Behavior, and Well-Being in Romantic Couples
Source: Front Psychol. 2021 Jan 18;11:622492. doi: 10.3389/fpsyg.2020.622492 (PMC7848131; doi:10.3389/fpsyg.2020.622492)
Supplement: Supplementary file 1 [file Table_1.DOCX]

Supplementary Table S1

*Parameter estimates from mixed models testing the effect of support provided on couple’s daily MVPA, positive and negative affect, and relationship satisfaction, adjusting for reports of support receipt.*

|  | *MVPA (in minutes)* | |  | *Positive Affect* | |  | *Negative Affect* | |  | *Relationship Satisfaction* | |
| --- | --- | --- | --- | --- | --- | --- | --- | --- | --- | --- | --- |
| Fixed effects | Estimate | *SE* |  | Estimate | *SE* |  | Estimate | *SE* |  | Estimate | *SE* |
| Intercept | 48.45*** | 2.36 |  | 0.58*** | 0.01 |  | 0.18*** | 0.01 |  | 0.70*** | 0.01 |
| Gender | 4.60^†^ | 2.46 |  | -0.02 | 0.02 |  | -0.03* | 0.02 |  | -0.02 | 0.02 |
| Time | 0.15 | 0.16 |  | -0.001 | <0.001 |  | 0.001 | 0.001 |  | 0.002* | 0.001 |
| Weekend | -2.96 | 2.06 |  | 0.005 | 0.006 |  | -0.03*** | 0.01 |  | 0.04*** | 0.01 |
| Wear Time (in hours) | 2.36*** | 0.40 |  | - | - |  | - | - |  | - | - |
| *WITHIN effects* |  |  |  |  |  |  |  |  |  |  |  |
| Own support *provision* (actor effect) | 2.25* | 1.03 |  | 0.01* | 0.003 |  | -0.01* | 0.003 |  | 0.01** | 0.004 |
| Partner’s support *provision* (partner effect) | 4.56*** | 0.86 |  | -0.001 | 0.003 |  | -0.001 | 0.003 |  | 0.01^†^ | 0.004 |
| Own support *receipt* (actor effect) | 3.18*** | 0.79 |  | 0.01** | 0.003 |  | -0.005 | 0.003 |  | 0.03*** | 0.004 |
| Partner’s support *receipt* (partner effect) | -1.05 | 0.82 |  | 0.002 | 0.003 |  | -0.002 | 0.003 |  | 0.01* | 0.003 |
| *BETWEEN effects* |  |  |  |  |  |  |  |  |  |  |  |
| Own support *provision* (actor effect) | 3.80 | 4.22 |  | -0.01 | 0.02 |  | 0.06** | 0.02 |  | -0.08** | 0.03 |
| Partner’s support *provision* (partner effect) | 13.40** | 4.08 |  | -0.01 | 0.02 |  | -0.02 | 0.02 |  | -0.07* | 0.03 |
| Own support *receipt* (actor effect) | -4.29 | 3.97 |  | 0.02 | 0.02 |  | -0.02 | 0.02 |  | 0.08** | 0.03 |
| Partner’s support *receipt* (partner effect) | -11.67** | 3.84 |  | 0.003 | 0.02 |  | 0.005 | 0.02 |  | 0.07* | 0.03 |
|  |  |  |  |  |  |  |  |  |  |  |  |
| Random effects (variances)^a^ |  |  |  |  |  |  |  |  |  |  |  |
| Level 2 (between-person) |  |  |  |  |  |  |  |  |  |  |  |
| Intercept | 394.60*** | 64.94 |  | 0.01*** | 0.002 |  | 0.01*** | 0.001 |  | 0.02*** | 0.003 |
| Gender | 401.67*** | 78.76 |  | 0.02*** | 0.004 |  | 0.02*** | 0.003 |  | 0.03*** | 0.004 |
| Time | 0.11 | 0.28 |  | <0.001** | <0.001 |  | <0.001^†^ | <0.001 |  | <0.001* | <0.001 |
| Weekend | 201.15*** | 56.93 |  | 0.001 | 0.001 |  | 0.001 | <0.001 |  | 0.002** | 0.001 |
| Wear Time (in hours) | 1.34 | 2.13 |  | - | - |  | - | - |  | - | - |
| Own support *provision* (actor effect) | 22.65* | 9.39 |  | - ^a^ | - ^a^ |  | - ^a^ | - ^a^ |  | <0.001* | <0.001 |
| Partner’s support *provision* (partner effect) | - ^a^ | - ^a^ |  | <0.001 | <0.001 |  | <0.001 | <0.001 |  | <0.001 | <0.001 |
| Own support *receipt* (actor effect) | - ^a^ | - ^a^ |  | <0.001^†^ | <0.001 |  | <0.001* | <0.001 |  | <0.001^†^ | <0.001 |
| Partner’s support *receipt* (partner effect) | 4.10 | 6.19 |  | <0.001 | <0.001 |  | <0.001 | <0.001 |  | -^a^ | -^a^ |
| Level 1 (within-person) |  |  |  |  |  |  |  |  |  |  |  |
| Residual | 736.34*** | 25.51 |  | 0.01*** | <0.001 |  | 0.01*** | <0.001 |  | 0.02*** | 0.001 |
| Autocorrelation | 0.01 | 0.03 |  | 0.21*** | 0.03 |  | 0.20*** | 0.03 |  | 0.20*** | 0.03 |

*Note*. For Model on MVPA, *N* = 97 (194) couples (individuals) with a maximum of 28 days, *n* = 2259 available days; for models on positive and negative affect and relationship satisfaction, *N* = 99 (198) couples (individuals) with a maximum of 28 days, *n* = 2181 available days. SE = standard error. Gender is coded as female = -0.5 and male = 0.5. ^a^ Due to non-convergence, not all random effects could be computed. ^†^p < .10, *p < .05, **p < .01, ***p<.001

Supplementary Table S2

*Parameter estimates from mixed models testing the effect of support provided on couple’s daily positive and negative affect, relationship satisfaction and MVPA, adjusting for time spent together*

|  | *MVPA (in minutes)* | |  | *Positive Affect* | |  | *Negative Affect* | |  | *Relationship Satisfaction* | |
| --- | --- | --- | --- | --- | --- | --- | --- | --- | --- | --- | --- |
| Fixed effects | Estimate | *SE* |  | Estimate | *SE* |  | Estimate | SE |  | Estimate | SE |
| Intercept | 48.55*** | 2.41 |  | 0.58*** | 0.01 |  | 0.17*** | 0.01 |  | 0.70*** | 0.01 |
| Gender | 3.31 | 2.45 |  | -0.02 | 0.02 |  | -0.03^†^ | 0.02 |  | -0.02 | 0.02 |
| Time | 0.11 | 0.15 |  | -0.002^†^ | 0.001 |  | 0.001 | 0.001 |  | 0.001 | 0.001 |
| Weekend | -2.39 | 2.10 |  | 0.01 | 0.01 |  | -0.03*** | 0.01 |  | 0.04*** | 0.01 |
| Wear Time (in hours) | 2.31*** | 0.40 |  | - | - |  | - | - |  | - | - |
| *WITHIN effects* |  |  |  |  |  |  |  |  |  |  |  |
| Own support *provision* (actor effect) | 3.82*** | 0.94 |  | 0.02*** | 0.003 |  | -0.01** | 0.003 |  | 0.03*** | 0.004 |
| Partner’s support *provision* (partner effect) | 4.58*** | 0.73 |  | 0.001 | 0.003 |  | -0.003 | 0.002 |  | 0.01*** | 0.004 |
| Daily time spent together | -0.05 | 0.08 |  | <0.001 | <0.001 |  | <-0.001 | <0.001 |  | 0.001*** | <0.001 |
| *BETWEEN effects* |  |  |  |  |  |  |  |  |  |  |  |
| Own support *provision* (actor effect) | -0.89 | 1.89 |  | 0.01 | 0.01 |  | 0.04*** | 0.01 |  | -0.01 | 0.01 |
| Partner’s support *provision* (partner effect) | 2.99 | 1.90 |  | -0.01 | 0.01 |  | -0.01 | 0.01 |  | 0.001 | 0.01 |
| Mean time spent together | -0.69 | 0.57 |  | 0.005^†^ | 0.003 |  | -0.004 | 0.003 |  | 0.01* | 0.003 |
|  |  |  |  |  |  |  |  |  |  |  |  |
| Random effects (variances)^a^ |  |  |  |  |  |  |  |  |  |  |  |
| Level 2 (between-person) |  |  |  |  |  |  |  |  |  |  |  |
| Male | 414.63*** | 67.63 |  | 0.01*** | 0.001 |  | 0.01*** | 0.001 |  | 0.02*** | 0.003 |
| Female | 421.60*** | 82.58 |  | 0.03*** | 0.004 |  | 0.02*** | 0.003 |  | 0.03*** | 0.004 |
| Time | 0.04 | 0.27 |  | <0.001** | <0.001 |  | <0.001^†^ | <0.001 |  | <0.001* | <0.001 |
| Weekend | 201.95*** | 56.82 |  | 0.001 | 0.001 |  | 0.001^†^ | <0.001 |  | 0.002* | 0.001 |
| Wear Time (in hours) | 1.46 | 2.14 |  | - | - |  | - | - |  | - | - |
| Own support *provision* (actor effect) | 24.96** | 9.45 |  | <0.001 | <0.001 |  | <0.001 | <0.001 |  | <0.001* | <0.001 |
| Partner’s support *provision* (partner effect) | - ^a^ | - ^a^ |  | <0.001 | <0.001 |  | <0.001 | <0.001 |  | <0.001 | <0.001 |
| Level 1 (within-person) |  |  |  |  |  |  |  |  |  |  |  |
| Residual | 746.49*** | 25.51 |  | 0.01*** | <0.001 |  | 0.01*** | <0.001 |  | 0.02*** | 0.001 |
| Autocorrelation | 0.005 | 0.03 |  | 0.22*** | 0.02 |  | 0.19*** | 0.03 |  | 0.20*** | 0.03 |

*Note*. For Model on MVPA, *N* = 97 (194) couples (individuals) with a maximum of 28 days, *n* = 2259 available days; for models on positive and negative affect and relationship satisfaction, *N* = 99 (198) couples (individuals) with a maximum of 28 days, *n* = 2181 available days. SE = standard error. Gender is coded as female = -0.5 and male = 0.5. ^a^ Due to non-convergence, not all random effects could be computed. ^†^p < .10, *p < .05, **p < .01, ***p<.001

Supplementary Table S3

*Parameter estimates from mixed models testing the effect of support provided on couple’s daily MVPA, positive and negative affect, and relationship satisfaction, adjusting for baseline socio-demographics and intervention group*

|  | *MVPA (in minutes)* | |  | *Positive Affect* | |  | *Negative Affect* | |  | *Relationship Satisfaction* | |
| --- | --- | --- | --- | --- | --- | --- | --- | --- | --- | --- | --- |
| Fixed effects | Estimate | *SE* |  | Estimate | *SE* |  | Estimate | *SE* |  | Estimate | *SE* |
| Intercept | 49.12*** | 3.03 |  | 0.57*** | 0.02 |  | 0.19*** | 0.01 |  | 0.71*** | 0.03 |
| Gender | 4.90^†^ | 2.52 |  | -0.02 | 0.02 |  | -0.03^†^ | 0.02 |  | -0.02 | 0.02 |
| Time | 0.10 | 0.15 |  | -0.002^†^ | 0.001 |  | 0.001 | 0.001 |  | 0.002^†^ | 0.001 |
| Weekend | -2.64 | 2.05 |  | 0.01 | 0.01 |  | -0.03*** | 0.01 |  | 0.05*** | 0.01 |
| Wear Time (in hours) | 2.28*** | 0.41 |  | - | - |  | - | - |  | - | - |
| *WITHIN effects* |  |  |  |  |  |  |  |  |  |  |  |
| Own support *provision* (actor effect) | 3.81*** | 0.94 |  | 0.02*** | 0.003 |  | -0.01** | 0.003 |  | 0.03*** | 0.004 |
| Partner’s support *provision* (partner effect) | 4.56*** | 0.72 |  | 0.002 | 0.003 |  | -0.003 | 0.003 |  | 0.01*** | 0.004 |
| *BETWEEN effects* |  |  |  |  |  |  |  |  |  |  |  |
| Own support *provision* (actor effect) | 0.28 | 1.83 |  | 0.01 | 0.01 |  | 0.04*** | 0.01 |  | -0.002 | 0.01 |
| Partner’s support *provision* (partner effect) | 4.32* | 1.85 |  | -0.01 | 0.01 |  | -0.01 | 0.01 |  | 0.01 | 0.01 |
| Intervention Group _(0=control, 1=intervention)_ | -1.01 | 4.05 |  | 0.03 | 0.02 |  | -0.03 | 0.02 |  | 0.02 | 0.03 |
| Relationship length (in years) | 0.03 | 0.21 |  | - | - |  | - | - |  | -0.002 | 0.001 |
| Age (in years) | -0.62** | 0.22 |  | - | - |  | - | - |  | - | - |
| BMI | -0.39 | 0.34 |  | - | - |  | - | - |  | - | - |
| Having kids _(0=no, 1=yes)_ | - | - |  | - | - |  | - | - |  | -0.03 | 0.03 |
|  |  |  |  |  |  |  |  |  |  |  |  |
| Random effects (variances)^a^ |  |  |  |  |  |  |  |  |  |  |  |
| Level 2 (between-person) |  |  |  |  |  |  |  |  |  |  |  |
| Intercept | 337.96*** | 56.85 |  | 0.01*** | 0.001 |  | 0.01*** | 0.001 |  | 0.02*** | 0.003 |
| Gender | 437.66*** | 86.64 |  | 0.03*** | 0.004 |  | 0.02*** | 0.003 |  | 0.03*** | 0.004 |
| Time | - ^a^ | - ^a^ |  | <0.001** | <0.001 |  | <0.001^†^ | <0.001 |  | <0.001* | <0.001 |
| Weekend | 199.57*** | 56.45 |  | 0.001 | 0.001 |  | 0.001^†^ | <0.001 |  | 0.002** | 0.001 |
| Wear Time (in hours) | 1.74 | 2.19 |  | - | - |  | - | - |  | - | - |
| Own support *provision* (actor effect) | 24.84** | 9.40 |  | <0.001 | <0.001 |  | <0.001 | <0.001 |  | <0.001* | <0.001 |
| Partner’s support *provision* (partner effect) | - ^a^ | - ^a^ |  | <0.001 | <0.001 |  | <0.001 | <0.001 |  | <0.001 | <0.001 |
| Level 1 (within-person) |  |  |  |  |  |  |  |  |  |  |  |
| Residual | 747.40*** | 24.74 |  | 0.01*** | <0.001 |  | 0.01*** | <0.001 |  | 0.02*** | 0.001 |
| Autocorrelation | 0.01 | 0.03 |  | 0.22*** | 0.02 |  | 0.19*** | 0.03 |  | 0.20*** | 0.03 |

*Note*. For Model on MVPA, *N* = 97 (194) couples (individuals) with a maximum of 28 days, *n* = 2259 available days; for models on positive and negative affect and relationship satisfaction, *N* = 99 (198) couples (individuals) with a maximum of 28 days, *n* = 2181 available days. SE = standard error. Gender is coded as female = -0.5 and male = 0.5. ^a^ Due to non-convergence, not all random effects could be computed. ^†^p < .10, *p < .05, **p < .01, ***p<.001
